# Whole Genome Sequencing Reveals Complex Evolution Patterns of Multidrug-Resistant *Mycobacterium tuberculosis* Beijing Strains in Patients

**DOI:** 10.1371/journal.pone.0082551

**Published:** 2013-12-06

**Authors:** Matthias Merker, Thomas A. Kohl, Andreas Roetzer, Leona Truebe, Elvira Richter, Sabine Rüsch-Gerdes, Lanfranco Fattorini, Marco R. Oggioni, Helen Cox, Francis Varaine, Stefan Niemann

**Affiliations:** 1 Molecular Mycobacteriology, Research Center Borstel, Borstel, Germany; 2 National Reference Center for Mycobacteria, Research Center Borstel, Borstel, Germany; 3 Dipartimento di Malattie Infettive, Parassitarie e Immunomediate, Istituto Superiore di Sanità, Rome, Italy; 4 Department of Genetics, University of Leicester, Leicester, United Kingdom; 5 Médecins sans Frontières, Cape Town, South Africa; 6 Centre of Infectious Disease Epidemiology & Research, University of Cape Town, Cape Town, South Africa; 7 Medical Department, Médecins sans Frontières, Paris, France; 8 Biomedizinische ForschungsgmbH, Vienna, Austria; 9 Department of Medical Microbiology, Virology and Hygiene, Universitätsklinikum Eppendorf, Hamburg, Germany; University of California, San Francisco, United States of America

## Abstract

Multidrug-resistant (MDR) *Mycobacterium tuberculosis* complex (MTBC) strains represent a major threat for tuberculosis (TB) control. Treatment of MDR-TB patients is long and less effective, resulting in a significant number of treatment failures. The development of further resistances leads to extensively drug-resistant (XDR) variants. However, data on the individual reasons for treatment failure, e.g. an induced mutational burst, and on the evolution of bacteria in the patient are only sparsely available. To address this question, we investigated the intra-patient evolution of serial MTBC isolates obtained from three MDR-TB patients undergoing longitudinal treatment, finally leading to XDR-TB. Sequential isolates displayed identical IS*6110* fingerprint patterns, suggesting the absence of exogenous re-infection. We utilized whole genome sequencing (WGS) to screen for variations in three isolates from Patient A and four isolates from Patient B and C, respectively. Acquired polymorphisms were subsequently validated in up to 15 serial isolates by Sanger sequencing. We determined eight (Patient A) and nine (Patient B) polymorphisms, which occurred in a stepwise manner during the course of the therapy and were linked to resistance or a potential compensatory mechanism. For both patients, our analysis revealed the long-term co-existence of clonal subpopulations that displayed different drug resistance allele combinations. Out of these, the most resistant clone was fixed in the population. In contrast, baseline and follow-up isolates of Patient C were distinguished each by eleven unique polymorphisms, indicating an exogenous re-infection with an XDR strain not detected by IS*6110* RFLP typing. Our study demonstrates that intra-patient microevolution of MDR-MTBC strains under longitudinal treatment is more complex than previously anticipated. However, a mutator phenotype was not detected. The presence of different subpopulations might confound phenotypic and molecular drug resistance tests. Furthermore, high resolution WGS analysis is necessary to accurately detect exogenous re-infection as classical genotyping lacks discriminatory power in high incidence settings.

## Introduction

Infection with strains of the *Mycobacterium tuberculosis* complex (MTBC), the causative agent of tuberculosis (TB), is responsible for approximately 1.4 million deaths annually [1]. Even more worrisome is the worldwide emergence of resistant, multidrug-resistant (MDR), or extensively drug-resistant (XDR) MTBC strains that pose a serious challenge for global TB control and make successful treatment difficult or impossible [1]. MDR strains are resistant to at least isoniazid (H) and rifampicin (R), the most effective anti-TB first-line drugs. XDR is defined as MDR with additional resistance to a fluoroquinolone, e.g. levofloxacin (Lfx), moxifloxacin (Mfx), ofloxacin (Ofx), plus resistance to any of the second-line injectable drugs capreomycin (Cm), amikacin (Am) or kanamycin (Km). Recently, the WHO estimated 650.000 cases (including 150,000 deaths) of MDR-TB with an estimated XDR-TB rate of 9% in 2010 [1]. 

Drug resistant TB infection can be caused by transmission of already resistant strains (primary resistance) or by selection of resistance conferring mutations during inadequate therapy (secondary resistance). Our own work [2] and a recent meta analysis [3] indicated that the success of MDR treatment is low in high incidence settings. Even during effective second-line treatment, the development of additional resistances led to XDR-TB in a significant number of cases. Transmission and acquisition of drug resistance appear to depend on the genetic background (phylogenetic lineage) of a strain population circulating in a particular region. Different genetic backgrounds might compensate for the fitness costs of acquired resistance leading to more efficient transmission of resistant strains or to rapid emergence of resistance in the course of the therapy due to higher mutation rates [4,5]. This notion is strongly supported by the fact that in areas with the highest proportions of MDR strains (mainly in Eastern Europe), MDR-TB has been shown to be strongly associated with infecting strains belonging to the Beijing lineage of the MTBC [6–9].

Following earlier studies on the occurence of *E.coli* [10,11] and *P. aeruginosa* [12] mutator phenotypes (with up to 1,000 fold increased mutation rates) in changing environmental conditions, de Steenwinkel and colleagues hypothesized a higher mutation rate for Beijing strains under antibiotic therapy. The authors showed that Beijing genotype strains exhibit significantly higher (1.6 × 10^−5^ to 5.4 × 10^−3^ versus 6.3 × 10^−8^ to 3.8 × 10^−4^, p = 0.003) mutation rates during experimental rifampicin treatment compared to strains of the East African Indian (EAI) lineage [5]. This observation was confirmed by Ford et al. who reported a more rapid acquisition of drug resistance *in vitro* for lineage 2 strains (comprising Beijing isolates) compared to lineage 4 strains, although on a much lower level [13]. While these studies pointed to a higher mutation rate of lineage 2 strains that might explain an enhanced resistance acquisition during standard therapy, earlier studies using fluctuation assays did not show an enhanced mutation capacity of Beijing strains [14]. 

Considering these controversial findings, direct investigations of the genome evolution of clinical isolates in transmission chains or in patients undergoing longitudinal therapy are desirable. Recent studies applying Next Generation Sequencing (NGS) technologies revealed that *M. tuberculosis* genomes are highly stable during human to human transmission and evolve with a mutation rate of approximately 0.5 single nucleotide polymorphisms (SNPs) per genome per year [15,16]. So far, only two studies have used NGS to analyze the genomes of serial isolates obtained from patients during longitudinal treatment [17,18]. Saunders et al. 2011 determined a very low mutation rate in a non-Beijing strain (personal communication) and concluded that its genome is relatively stable within the host showing lower variability as suggested by studies on emergence of drug resistance and hypermutability [5,13]. However, Sun et al. showed for serial isolates of three patients infected with Beijing strains that the evolution in the patient might be more complex and the population structure within a patient can be diverse [18]. The authors identified 8-41 SNPs per sample, the majority of which (82.7%) were only found at frequencies below 20%, including four to five different resistance conferring variants [18]. This might go in line with higher mutation rates of Beijing strains in general leading to more complex evolutionary patterns and faster acquisition of drug resistance. The authors also used specific analysis tools to define low level variants (5%) that explained a significant number of variations in their study. 

**Figure 1:**
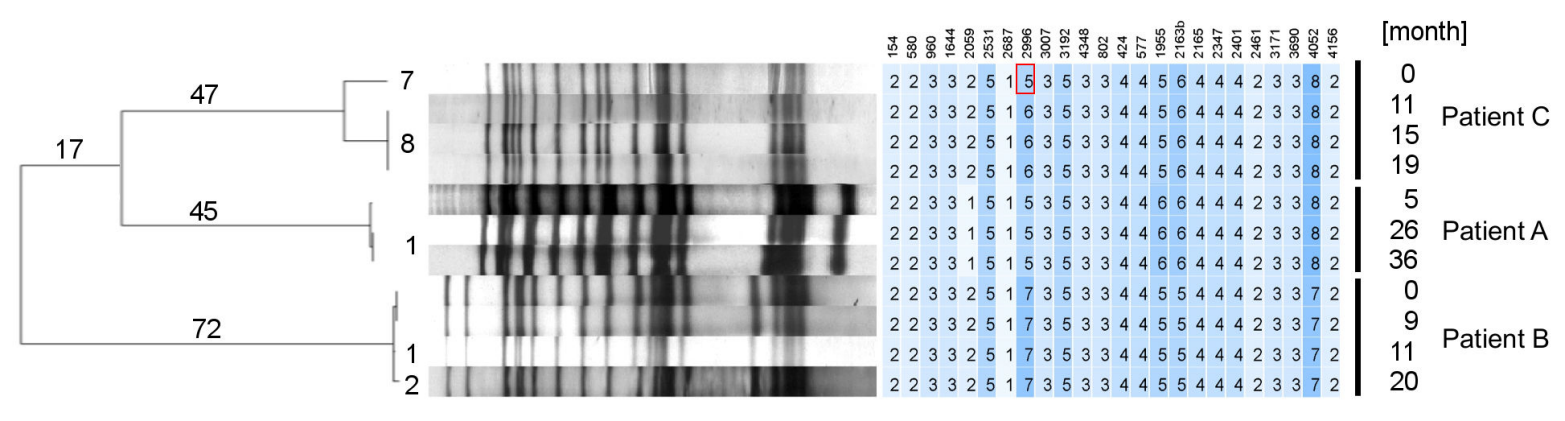
Genotyping data of all analyzed patient derived isolates. Unrooted UPGMA tree based on 289 concatenated single nucleotide polymorphisms (SNPs) differentiating all analyzed W-Beijing isolates illustrated with corresponding IS*6110* RFLP and 24-loci MIRU-VNTR patterns. Vertical lines indicate patient derived isolates. Known resistance conferring SNPs were excluded.

To follow up on these findings, we investigated whether a higher mutation rate is a general Beijing genotype characteristic that holds true for Beijing strains from other areas with high rates of MDR-TB. Therefore, we carried out whole genome sequencing (WGS) of serial isolates derived from three MDR-TB patients infected with Beijing strains undergoing long-term treatment of up to 32 months. All isolates belonged to the W-Beijing family and were closely related to strain types that were described as successful MDR clones in Karakalpakstan (Uzbekistan) [7], Kazakhstan [6], Turkmenistan [7] and Abkhazia (Republic of Georgia) [8] (see Figure 1). Special attention was given to the way mutations occurred, e.g. in a stepwise manner related to the emergence of resistance to additional drugs as suggested by Saunders and colleagues [17], or by mutational burst during antibiotic treatment. A transient or stable MTBC mutator phenotype, caused by higher replication failure rates, might have substantially increased mutation rates (10 to 1,000 fold increased) as indicated by previous studies [5,10]. This in turn would co-select numerous mutations, e.g. in non-coding regions and non-functional mutations that hitchhike together with new resistance mutations. To investigate this question we screened the genomes for hitchhiking mutations that are likely to emerge in a hypermutable clone.

## Materials and Methods

### Patient derived isolates

The three patients (A, B, C) were part of epidemiological studies in Germany and Eastern Europe. Standardized protocols for mycobacterial growth, DNA extraction, drug susceptibility testing (DST), RFLP fingerprint, 24-loci MIRU-VNTR and spoligotyping can be found in respective publications (Patient A – Germany [19], Patient B – Abkhazia (Republic of Georgia) [8,20], Patient C – Karakalpakstan (Uzbekistan) [2]). DNA was isolated from subcultures grown on Löwenstein-Jensen slants from sputum culture without single colony passage to allow for investigation of the population diversity within one isolate. 

### Identification of Polymorphisms

Genomic DNA of selected isolates was sequenced on Illumina instruments at GATC Biotech (Konstanz, Germany). For Patient A and C derived isolates we used 72 bp paired end reads, and for Patient B 46 bp paired end reads, respectively. Reads were mapped to the *M. tuberculosis* H37Rv genome (GenBank ID: NC_000962.2) employing the exact alignment program SARUMAN [21]. For all isolates, over 96% of the *M. tuberculosis* H37Rv genome was covered with at least one read. Paired end reads from all analyzed isolates were deposited in the NCBI sequence read archive (SRA) and can be found under the accession number SRA060746.

Genomic regions featuring high GC content or repetitive elements (e.g. PE/PPE/PGRS gene families, ESAT-6 like, *lppA*, *lppB*, *pks12*) offer a serious challenge to WGS and data analysis, which is often indicated by a low variant frequency (<50%). To avoid the inclusion of false positives in our data set and to detect the fixation of possible beneficial variants within the population, we extracted polymorphisms from mapped reads by in house Perl scripts using rather conservative quality threshold levels, i.e. a minimum coverage of ten reads and a minimum allele frequency of 75%. Detected polymorphisms of all analyzed isolates were combined and majority nucleotides for positions without a variant call were inferred from mapping data (Table S7 in File S1). We screened serial patient derived isolates subsequently for fixed mutations that occurred during the treatment with at least 90% allele frequency and adjusted coverage thresholds, and one outlier allowed. All polymorphisms differentiating serial isolates and genes associated with acquired drug resistances were further validated by Sanger sequencing using an ABI3130xl Sequencer. In addition, we applied VarDetect (vers. 200601251500) [22] to analyze peak height ratios of heterogeneous SNPs in order to estimate the frequency of different dominating subpopulations in all serial patient derived isolates (see also Method S1). 

## Results

Based on our large in house database comprising IS*6110* RFLP and spoligotyping patterns of MTBC strains analyzed in previous studies, we selected three TB patients infected with W-Beijing strains exhibiting a massive acquisition of additional drug resistances during treatment, finally leading to XDR-TB. In each case, exogenous re-infection was excluded in the underlying study by identical IS*6110* fingerprint patterns of the serial isolates (Figure 1). The three patients have been enrolled in studies performed in Germany [19], Abkhazia (Republic of Georgia) [20], and Karakalpakstan (Uzbekistan) [2] . 

**Figure 2:**
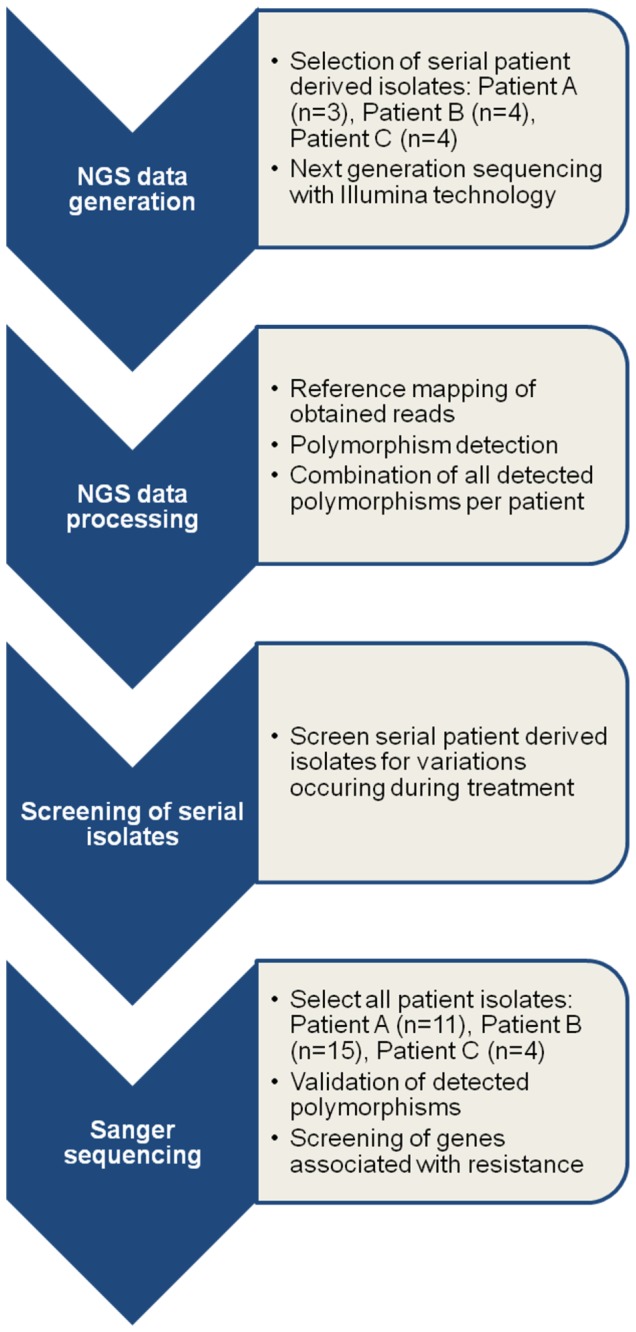
Workflow. Next generation sequencing (NGS) data can be found under the accession number SRA060746. NGS reads were mapped with SARUMAN (Blom et al. 2011) to the H37Rv reference strain (GenBank ID: NC_000962.2). Variant detection was carried out with in house Perl scripts, followed by screening for high frequency polymorphisms that occurred during treatment. Finally, we validated all detected polymorphisms and genes associated with acquired resistances in each patient with Sanger sequencing in all available serial isolates.

Three isolates from Patient A and four isolates from Patient B and C, respectively, covering the whole treatment period, have been subjected to WGS. Obtained reads were processed with our in house software pipeline described in Material and Methods and Figure 2. SNPs and small deletions of subpopulations predominating in selected isolates and genes associated with resistance acquisition were further examined by Sanger sequencing in up to 15 serial isolates and then linked to the acquisition of phenotypic resistance to first and second-line drugs. Furthermore, we applied VarDetect [22] to calculate peak height ratios for heterogeneous SNPs and to roughly estimate the nucleotide mixture and the frequency of each subpopulation, respectively, in all patient derived isolates. 

### Intra-host genome evolution of Patient A and B isolates was restricted to acquisition of drug resistance conferring mutations

Patient A was dually infected with an H and streptomycin (S) resistant non-Beijing strain and a MDR W-Beijing strain that was resistant to H, R, S and ethambutol (E). The initial treatment was effective against the non-Beijing strain but inappropriate for the W-Beijing type strain. Outdated DST data led to the development of resistance to six additional drugs (including pyrazinamide (Z), prothionamide (Pto), PAS, Am, Cm, Lfx) after clearance of the non-Beijing strain, resulting in a “hyper-resistant” phenotype after 32 months of MDR-TB treatment [19].

**Figure 3:**
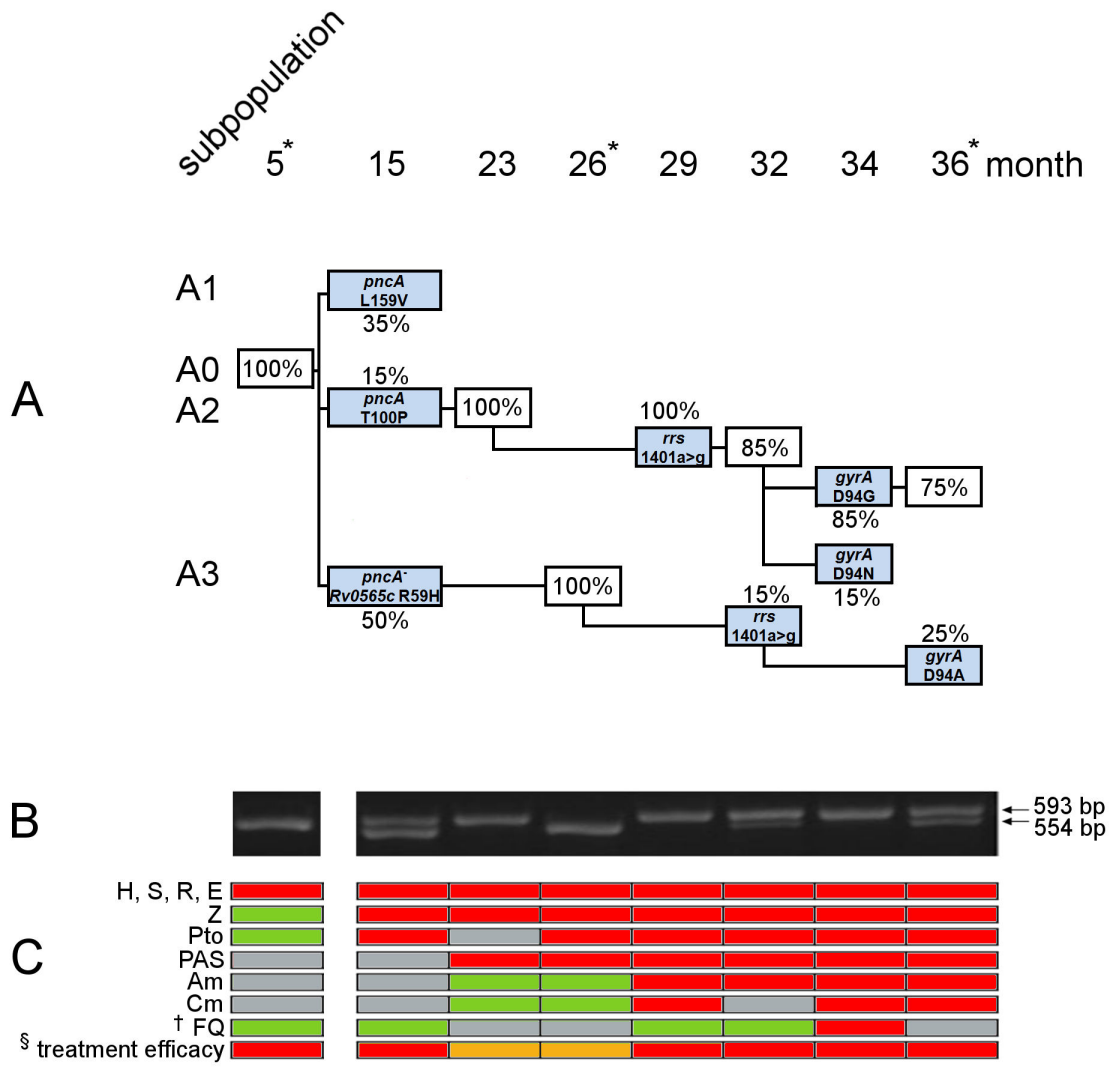
Evolution and competition of Patient A derived clones during MDR-TB therapy. * Isolates selected for whole genome sequencing (WGS). † Isolate on month 15 was tested susceptible to ofloxacin, isolates on month 29 was tested susceptible to ciprofloxacin, isolate on month 34 was tested resistant to levofloxacin. § Treatment efficacy is annotated as green=more than two effective drugs applied at the given time point, orange=two drugs being active, red=only one drug active or treatment failure. A) Model of intra-patient evolution and competition during 32 months of MDR-TB therapy of different W-Beijing subpopulations (A1, A2, A3) in Patient A originating from the initial clone A0. Newly detected clones are colored in light blue. Peak height ratios calculated by VarDetect [22] of corresponding chromatograms were used to estimate the frequency of predominating subpopulation in all serial patient derived isolates. B) Multiplex-PCR analysis of Patient A derived isolates targeting a 3.1 kb deletion affecting *Rv2042* to *Rv2045* (including *pncA*) showing a mixture of clones after 15 month of MDR-TB therapy. The larger fragment (593 bp) represents the presence of that region; the 554 bp fragment is the product of primers flanking the large deletion. C) Resistogram of phenotypic DST results. Red=resistant, green=susceptible, grey=no data. (A detailed description of genotypic and phenotypic drug resistance acquisition of Patient A derived serial isolates, as well as applied regimens are presented in Table S2 in File S1).

In comparison to H37Rv, we identified 1072 polymorphisms by WGS, four of which were variable among the three selected serial isolates, including a large deletion (3.1kb) in one isolate (Table S1 in File S1). With respect to previously described drug resistance associated mutations, all isolates had the following variants: S315T in the gene *katG* known to confer resistance to H [23], K43R in the gene *rpsL* shown to confer resistance to S [24], and M306I in the gene *embB* linked with resistance to E [25] (Table S1 in File S1). No SNP was present in the 81bp “hot-spot” region of the gene *rpoB* ranging from codons 426-452 in H37Rv (GenBank ID: NC_000962.2), but all isolates carried the mutations V170F in *rpoB* and V483A, E1092D in *rpoC*. The mutation V483A in *rpoC* has been described to compensate for fitness costs of classical *rpoB* “hot-spot” mutations causing resistance to R [26]. The mutation E1092D in *rpoC* was also found in R susceptible Beijing strains [27], thus most likely representing a phylogenetic rather than a resistance causing variant. Hence, the mutation V170F in *rpoB* is the most likely cause of resistance to R in this Beijing strain that might have acquired enhanced virulence by the compensatory mutation V483A in *rpoC*. All isolates exhibited the nonsense mutation Q215* in the gene *ethA* (monooxygenase) previously described to be involved in resistance to Pto [28,29]. However, phenotypic resistance to Pto was not detected till month 15 (Figure 3C) and coincided with the mutation R59H in *Rv0565c*, encoding a putative monooxygenase.

To further examine the exact course of genome evolution, we analyzed the four detected polymorphisms differentiating selected serial isolates and genes associated with resistance acquisition to Z, Pto, PAS, Am, Cm, Lfx (*pncA, ethA, thyA, rrs, gyrA*, respectively) by Sanger sequencing for eleven Patient A derived isolates (Table S2 in File S1). This analysis revealed additional heterogeneous SNPs indicating the simultaneous presence and competition of three subpopulations (A1, A2 and A3) emerging in Patient A and coinciding with resistance to Z and Pto (Figure 3A). Nucleotide variants, characterizing each subpopulation, were found for the genes *pncA* associated with resistance to Z [24,30] and *Rv0565c*. The clone *pncA* L159V (A1) was found in one patient derived isolate at month 15 only (Figure 3A). In contrast, clones *pncA* T100P (A2) and *pncA* ^*-*^ (A3), exhibiting a 3.1kb deletion affecting *Rv2042* to *Rv2045* (including *pncA*) in combination with R59H in *Rv0565c*, were detected by PCR analysis until month 36 (Figure 3B). During the course of the treatment, clones *pncA* ^*-*^ (A3) and *pncA* T100P (A2) independently acquired the mutation 1401a>g in the gene *rrs* conferring cross-resistance to Am and Cm [24,31]. Subsequently, 34 months after starting the treatment, clone *pncA* T100P (A2) acquired either the mutation D94G or D94N in the gene *gyrA*, while clone *pncA* ^*-*^ (A3) acquired the mutation D94A in the gene *gyrA* (Figure 3A), all leading to resistance to Lfx [24,32].

**Figure 4:**
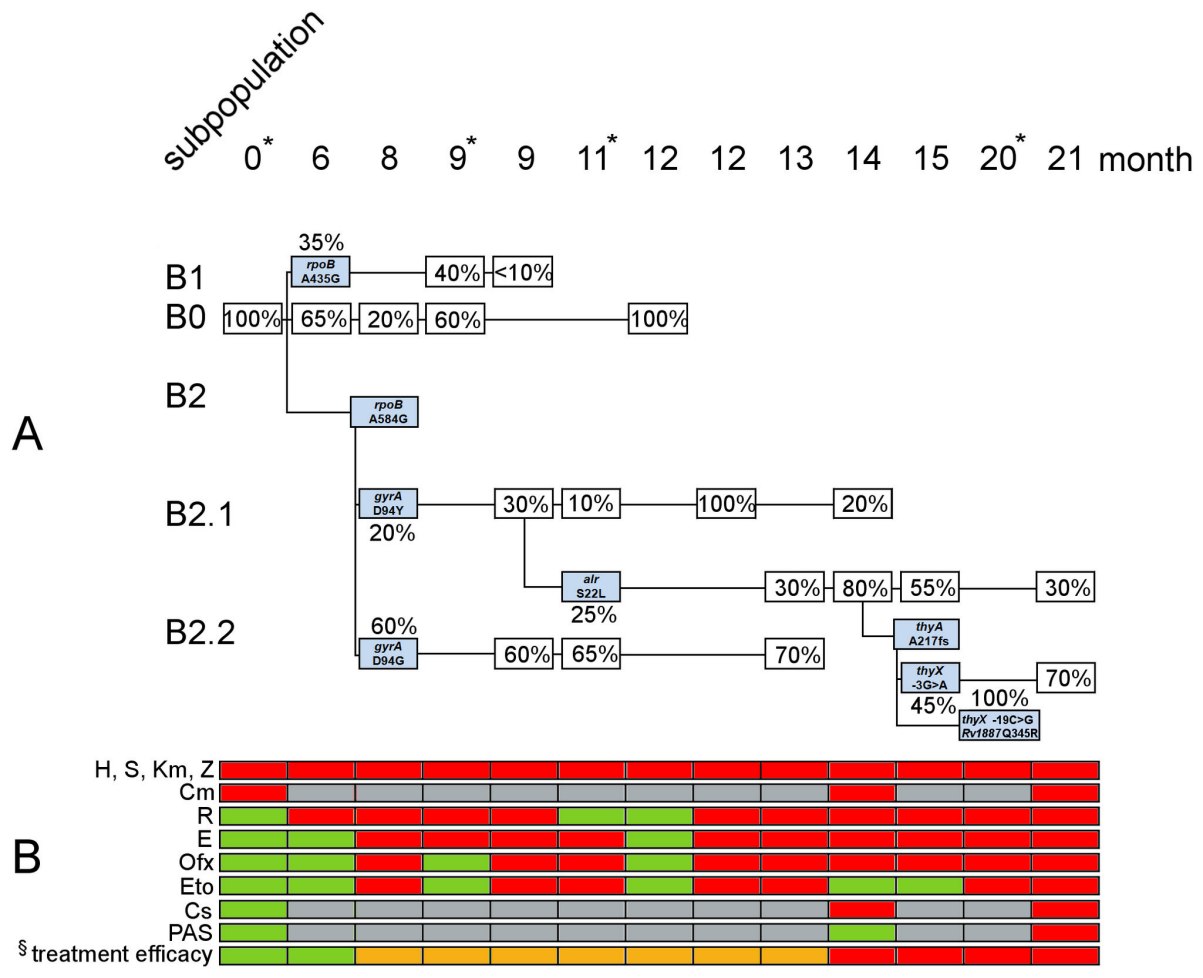
Evolution and competition of Patient B derived clones during MDR-TB therapy. * Isolates selected for whole genome sequencing (WGS). § Treatment efficacy is annotated as green=more than two effective drugs applied at the given time point, orange=two drugs being active, red=only one drug active or treatment failure. A) Model of intra-patient evolution and competition during 21 months of MDR-TB therapy of different W-Beijing subpopulations (B1, B2, B2.1, B2.2) in Patient B originating from the initial clone B0. Newly detected clones are colored in light blue. Peak height ratios calculated by VarDetect [22] of corresponding chromatograms were used to estimate the frequency of predominating subpopulation in all serial patient derived isolates. B) Resistogram of phenotypic DST results. Red=resistant, green=susceptible, grey=no data. (A detailed description of genotypic and phenotypic drug resistance acquisition of Patient B derived serial isolates, as well as applied regimens are presented in Table S4 in File S1).

Patient B was initially enrolled in a prospective cross-sectional study between 2003 and 2005, which targeted determinants of drug-resistant tuberculosis in Abkhazia (Republic of Georgia) [8,20]. The patient was integrated into the study as non-MDR case, while the baseline isolate was tested resistant to H, S and Km. During the course of first-line antibiotic treatment (13 months) comprising a regimen of H, R, E and Z, additional resistances to R and E were detected and Patient B was classified as MDR case. According to the latest DST results the treatment was changed to ethionamide (Eto), Cm, Ofx, Cycloserine (Cs) and PAS (month 0 of MDR therapy). During the course of another 21 months of MDR treatment, 15 serial isolates were obtained with alternating episodes of R, E, Ofx, Eto resistant and susceptible isolates. All isolates were initially tested susceptible to Cm, Cs and PAS, respectively. Based on the sequencing results, DST was repeated for selected isolates and we could confirm cross resistance between Km and Cm, as well as resistance acquisition to Cs and PAS during treatment (Figure 4B). 

WGS analysis identified 1575 polymorphisms compared to H37Rv, six of which were variable among the four selected isolates (Table S3 in File S1). All isolates featured the mutation *katG* (S315T) in combination with *inhA* (-15C>T) conferring resistance to H [23] and low level resistance to Eto [33,34], *rpsL* (K43R) conferring resistance to S [24], *pncA* (L151S) associated with resistance to Z [24,30] and *rrs* (1401a>g) conferring cross resistance to Km and Cm [24,31] respectively. Furthermore, we found the mutation L452P in the “hot-spot” region of *rpoB* that has been controversially described to be associated with low level and high level resistance to R [35] and the mutation G406D in the gene *embB*, shown to confer resistance to E [36]. Drug resistance to E was not detected in the first two isolates. However, resistance to E was found by susceptibility testing of the following isolates.

Again we screened all Patient B derived serial isolates (n=15) by Sanger sequencing for the six mutations identified in selected specimens analyzed by WGS plus genes associated with acquired resistance to R, E, Ofx, Eto, Cs, PAS (*rpoB, embB, gyrA, ethA, alr* and *thyA*) (Table S4 in File S1).

Overall eight SNPs and one 1bp frameshift mutation emerged during treatment in correlation with acquisition of additional resistances and associated with the presence of five subpopulations (B0, B1, B2, B2.1, B2.2). The subpopulations were initially distinguished by different mutations in *rpoB*: A435G (B1) and A584G (B2), both correlating with an R resistant phenotype (Figure 4A, 4B). The MDR baseline R susceptible clone (B0) could still be detected on month twelve exhibiting no additional mutations. Clone *rpoB* A584G (B2) was identified on month eight for the first time and later independently acquired either the mutation D94Y (B2.1) or D94G (B2.2), respectively, in *gyrA* conferring resistance to Ofx [32]. The clone B2.1 exhibiting the combination *rpoB* A584G and *gyrA* D94Y further acquired the mutation S22L in the gene *alr* (*Rv3423c*, D-alanine racemase) on month eleven and was the only clone found in isolates after 13 months of MDR-TB therapy, thus outcompeting all other clones (Figure 4A). Subsequently, clone B2.1 further acquired mutations in the genes *thyA* (*Rv2764c*, thymidylate synthase), *thyX* (*Rv2754c*, thymidylate synthase) and *Rv1887* (hypothetical protein). The mutation S22L in the gene *alr* coincided with a Cs resistant phenotype, whereas a frame shift mutation in *thyA* was linked with PAS resistance [37]. Interestingly, the disruption of the *thyA* gene by a 1 bp deletion (A217fs) was linked with a mutation in the upstream region of *thyX* (-3G>A). On month 20 of MDR therapy we detected another descendant of B2.1 carrying the mutations -19C>G upstream of *thyX* (instead of -3G>A) in combination with Q245R in the gene *Rv1887*, representing another clone possibly compensating for the loss of ThyA (Figure 4A). 

### Whole Genome Sequencing Revealed an Exogenous re-Infection with an XDR Strain in Patient C

Patient C was selected from an MDR-TB treatment cohort of 87 patients treated in Karakalpakstan (Uzbekistan). In the initial study, we investigated the development of Ofx resistance and extensively drug-resistant tuberculosis during MDR-TB treatment and controlled for exogenous re-infection by IS*6110* RFLP typing of the serial isolates obtained in case of treatment failure [2].

**Figure 5:**
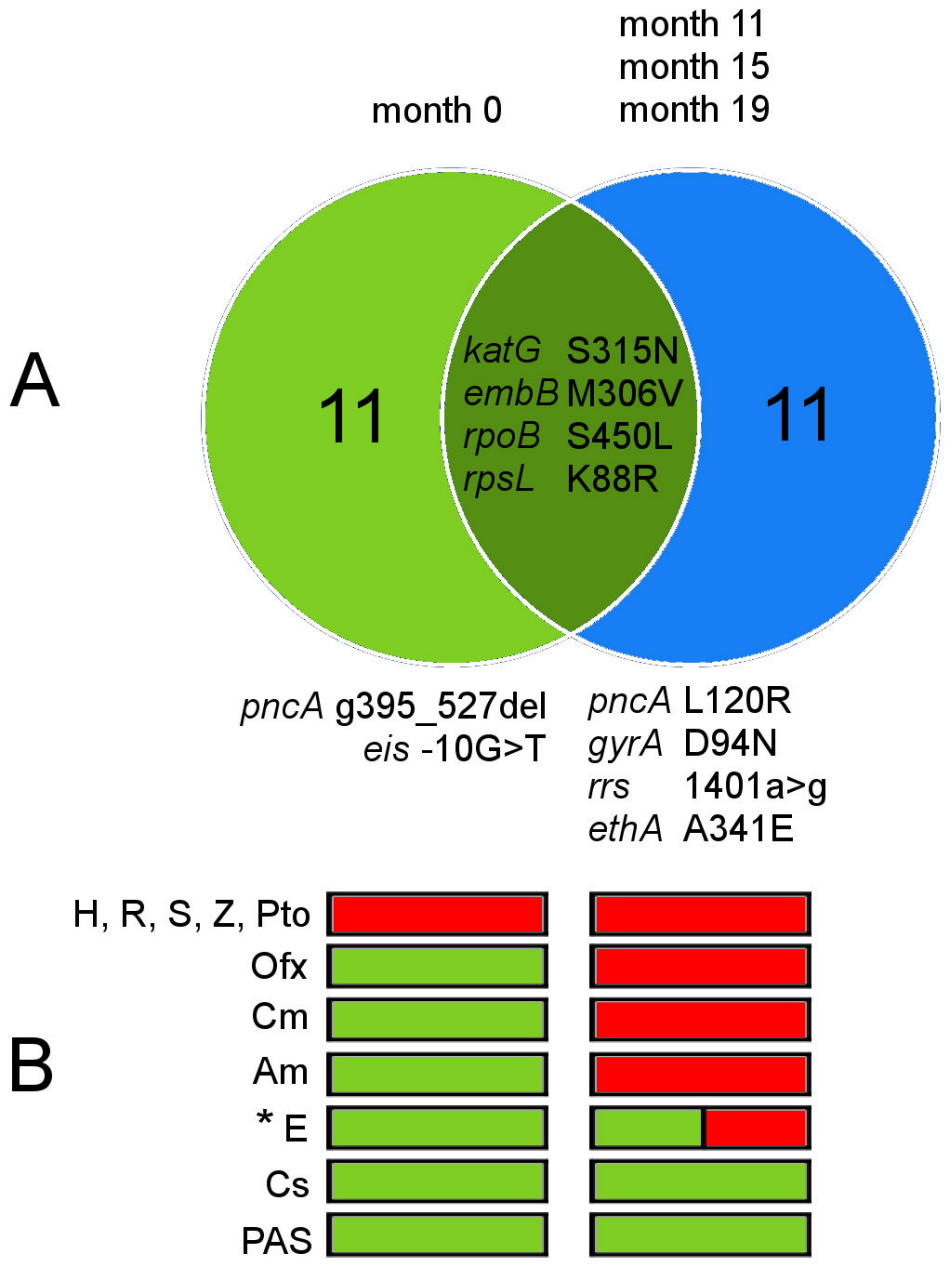
Exogenous re-infection with an XDR strain observed in Patient C. * Isolate on month 11 was testest susceptible to ethambutol (E), the following isolates on month 15 and 19 were tested phenotypically resistant; all isolates exhibit the mutation *embB* M306V. A) Venn diagram showing that while the baseline isolate (month 0) and follow up isolates (month 11-19) share common drug resistance mutations and identical IS*6110* RFLP fingerprints (Figure 1) both are clearly differentiated by eleven unique polymorphisms including additional drug resistance conferring genes. B) Resistogram of phenotypic DST results. Red=resistant, green=susceptible.

The baseline isolate was tested phenotypically resistant to H, R, S, Z and Pto. After eleven months of MDR-TB therapy, the isolate (month 11) was also tested resistant to Ofx, Cm and Am, thus classifying the isolate as XDR. On month 15 after starting the treatment, the patient derived isolate was tested resistant to E. The DST pattern was confirmed by a final isolate four months later (Figure 5B). All four isolates exhibited the same IS*6110* and spoligotype pattern, as well as identical mutations conferring resistance to H (*katG* S315N), E (*embB* M306V), R (*rpoB* S450L) and S (*rpsl* K88R) (Figure 5A, Table S5 in File S1), suggesting treatment failure with development of resistance to four (respectively three, when considering the possibility that isolates of month 0 and 11 could already have been resistant to E) additional antibiotics during 15 months of MDR-TB therapy.

In contrast to the findings in patients A and B showing a low variability and stepwise acquisition of few SNPs over time, we identified eight SNPs and three deletions unique to the initial isolate and eleven SNPs unique to the following isolates (Figure 5A, Table S5 and S6 in File S1), clearly separating the latter three isolates (month 11-19) from the baseline isolate. All three isolates obtained after month 11 exhibited no further polymorphisms and had identical SNP profiles with known drug resistance conferring mutations that coincided with an XDR phenotype (Figure 5A, 5B), e.g. the mutation D94N in the gene *gyrA* conferring resistance to Ofx [31,32], and the mutation 1401a>g in the gene *rrs* linked with Am and Cm cross resistance [24,31]. 

Likewise, mutations associated with resistance to Z [24,30] were different between baseline (*pncA* g.395_527del) and follow up isolates (*pncA* L120R), further contradicting an evolutionary link between the latter isolates and the baseline isolate (Figure 5A). Based on this data, we concluded that Patient C was re-infected with an XDR strain exhibiting the same IS*6110* pattern as the first MDR strain that was successfully treated by the applied MDR regimen [2]. This notion was supported by MIRU-VNTR typing performed later that revealed a difference in one out of 24 MIRU-VNTR loci (Figure 1).

## Discussion

In this study, we used WGS to determine the genome evolution of W-Beijing strains under longitudinal MDR-TB treatment of up to 32 months. Despite constant exposure to antibiotic stress during the whole treatment period, we could not find any indications for a burst of mutations e.g. by induction of erroneous or loosened replication control in the isolates investigated. Such a mutator type is expected to acquire a considerable number of additional mutations, e.g. non-functional mutations or intergenic mutations co-segregating with new drug resistance conferring mutations. However, nearly all mutations that were fixed in the population had a clear association with drug resistance mechanisms and occurred in a stepwise manner in strict correlation with the acquisition of new phenotypic resistances (see Table 1). This gradual acquisition of resistance conferring mutations and the absence of hitchhiking mutations contradicts the hypothesis of a stress induced mutational burst as proposed by others [5,38].

**Table 1:** Polymorphisms associated with drug resistance observed in this study.

Gene	Position**^*a*^**	SNP	Substitution	Associated resistance**^*b*^**	In combination with	Found in
*gyrA*	7,581	G>T	D94Y	FQ		Patient B
	7,581	G>A	D94N			Patient A
	7,582	A>G	D94G			Patient A, B
	7,582	A>C	D94A			Patient A
*rpoB*	760,314	G>T	V170F	R	*rpoC* V483A	Patient A
	761,110	A>G	A435G		*rpoB* L452P	Patient B
	761,155	C>T	S450L		*rpoC* H525N	Patient C
	761,557	C>G	A584G		*rpoB* L452P	Patient B
*rpsL*	781,687	A>G	K43R	S		Patient A, B
	781,822	A>G	K88R			Patient C
*rrs*	1,473,246	a>g	1401	Km/Am/Cm		Patient A, B, C
*katG*	2,155,168	C>G	S315T	H		Patient A
	2,155,168	C>G	S315T	H	*fabG1/inhA* -15C>T	Patient B
	2,155,168	C>T	S315N			Patient C
*pncA*	2,288,767	G>C	L159V	Z		Patient A
	2,288,790	A>G	L151S			Patient B
	2,288,883	A>C	L120R			Patient C
	2,288,944	T>G	T100P			Patient A
			g.395_527del			Patient C
			*Rv2042c*_*Rv2045c*del			Patient A
*eis*	2,715,342	C>T	-10	Km		Patient C
*thyA*	3,073,821	delG	A217fs	PAS	thyX -19C>G, -3G>A	Patient B
*alr*	3,841,356	G>A	S22L	Cs		Patient B
*embB*	4,247,429	A>G	M306V	E		Patient C
	4,247,431	G>C	M306I			Patient A
	4,247,730	G>A	G406D			Patient B
*ethA*	4,326,831	G>A	Q215*	Pto/Eto	*Rv0565c* R59H	Patient A
	4,326,452	G>T	A341E			Patient C

^a^ H37Rv genome (GenBank ID: NC_000962.2)

^b^ FQ=fluoroquinolones, R=rifampicin, S=streptomycin, Km=kanamycin, Cm=capreomycin, H=isoniazid, Z=pyrazinamide, PAS=para-aminosalicylic acid, Cs=cycloserine, E=ethambutol, Eto=ethionamide, Pto=prothionamide

In line with previous studies [18,39,40], our data demonstrate the longitudinal co-existence of different clonal subpopulations within one patient. We observed a complex clonal population structure with a variety of subpopulations displaying unique resistance conferring mutation profiles as well as a different composition of predominant subpopulations in distinct isolates taken at the same time point. Target sequencing of up to 15 serial isolates of the whole treatment period extended these findings and revealed the longitudinal parallel evolution of few dominating clones within one patient. This intra-patient diversity is likely to influence the performance of molecular and phenotypic drug resistance tests and needs to be considered for interpretation of routine diagnostic results [41,42]. New NGS based diagnostic algorithms need to consider that the presence of mixed populations in MDR-TB patients might be the normal situation rather than the exception, which poses special challenges for NGS based resistance diagnostics as those algorithms must be optimized to unravel distinct subpopulations.

The intra-patient evolution observed in patients A and B was characterized by several phases of ineffective drug regimens during which clonal competition drove the selection towards highly resistant phenotypes that outcompeted the less resistant or less fit variants. This evolutionary process led to the acquisition of additional resistance mutations and/or possible compensatory mutations, e.g. as found in the upstream region of *thyX* (-19C>G, -3G>A) in PAS resistant isolates in Patient B. Importantly, these mixed populations not only comprised compositions of susceptible and resistant alleles that might be expected when resistant cells are selected and replace susceptible ancestors, but they also demonstrated the parallel selection and evolution of different resistant subpopulations. This underlines the ability of MTBC strains to adapt and evolve within the patient. However, more studies are necessary to answer the intriguing question of how the phylogenetic background of clinical isolates can influence treatment outcome.

Our data demonstrated that second-line drugs used for treatment are indeed acting on MDR strains by exerting an evolutionary pressure that selects for drug resistance conferring mutations. For the first-line drug rifampicin, one interesting question is the importance of mutations outside the *rpoB* “hot-spot” region (codons 426-452, H37Rv annotation), which is usually interrogated by molecular resistance assays, for the development of resistance. For Patient B, our data indicated a stepwise acquisition of resistance to rifampicin that involved the initial acquisition of a probable low level resistance mutation (*rpoB* L452P) [35], followed by additional *rpoB* mutations: A435G (low level resistance) [43] and A584G (not reported so far), which finally conferred high level resistance to rifampicin. Furthermore, we found a yet unknown mutation V170F in the gene *rpoB*, in combination with the mutation V483A in the gene *rpoC*, suggested to be a potential compensatory mutation [26], which might explain the transmissibility of the MDR strain that infected Patient A [19]. 

Molecular mechanisms for the oral bacteriostatic second-line drugs PAS and Cs are only partly understood [24]. A previous study suggested that mutations in the gene *thyA* play a role in resistance development to PAS [37]. We could link a frameshift mutation in *thyA* (A217fs) to the emergence of PAS resistance in Patient B. This is in line with a recent report demonstrating that the absence of functional ThyA confers resistance to PAS [44]. Interestingly, mutations in the upstream region of *thyX* (-19C>G, -3G>A) emerged in the ThyA defective isolates. We suggest that these potential *thyX* promoter mutations compensate for the likely loss of function of ThyA. This notion is supported by findings determined in a recent study from Fivian-Hughes et al. [44]. The authors showed that ThyA and ThyX are different unrelated types of thymidylate (dTMP) synthases; while deletion of *thyA* conferred resistance to PAS, ThyX most likely compensated the loss of ThyA with a low dTMP synthase activity [44]. Thus, up-regulation of ThyX might represent an important compensatory mechanism contributing to fitness levels of PAS resistant strains. 

Furthermore, our analysis linked resistance to cycloserine to mutations in the gene *alr* (S22L). This is in line with reports suggesting the mycobacterial alanine racemase (Alr) as target for Cs [24,45] and overexpression experiments of the D-alanine racemase that led to Cs resistance in *Mycobacterium smegmatis* [46]. Cross resistance to the prodrugs ethionamide (Eto) and prothionamide (Pto) was suggested to be mediated by alterations in the gene *ethA* (monooxygenase) [28] or by up-regulation of the target protein InhA [33,34]. The data obtained for Patient A isolates let us assume that the disruption (Q215*) of *ethA* alone did not confer resistance to Pto. As suggested previously [29], the activation of Eto and Pto may include additional enzymes. Here, our data indicated the involvement of the acquired mutation R59H in the putative monooxygenase *Rv0565c* that coincided with resistance acquisition to Pto (Figure 3A, 3B). Ambiguous DST results for Eto in Patient B derived isolates are most likely the result of low level resistance conferred by the mutation *fabG1/inhA* -15 C>T.

We are confident that we identified an exogenous re-infection of a MDR patient with an XDR strain indistinguishable by IS*6110* fingerprint patterns and with identical resistance mutations to four first-line drugs. However, a mixed infection at baseline cannot be completely ruled out by the data obtained in the study. The XDR clone might have been absent in the baseline specimen by chance or only been present at very low frequency (not detectable by the applied 1% proportion method for DST). Importantly, we can exclude the clonal relatedness of both strains, and thereby highlight the risk of XDR-TB transmission in high incidence MDR-TB regions. Recent studies applying WGS on outbreak isolates defined the expected variations between clinical isolates from confirmed transmission chains to a maximum of five SNPs [15,16]. In contrast, baseline and latter isolates obtained from Patient C were distinguished each by eleven unique polymorphisms, and also displayed a single locus variant in the 24-loci MIRU-VNTR patterns. Early WGS based analysis of the genomes of closely related Beijing isolates already demonstrated that standard genotyping methods obviously lack discriminatory power. This makes the interpretation of molecular epidemiological studies on isolates from Eastern European high incidence settings, applying only such markers, rather difficult [47]. These observations have clear consequences for the interpretation of molecular epidemiological studies especially in high incidence areas and suggest that the analysis of ongoing transmission by standard molecular epidemiological techniques may establish false links between patients. This might lead to an overestimation of ongoing MTBC transmission and underestimation of the complexity of the pathogen population [47]. 

## Conclusion

Our results showed that the intra-patient evolution of W-Beijing strains was characterized by a stepwise acquisition of resistance conferring mutations selecting for the most resistant phenotype during inadequate MDR-TB therapy. This incremental process without an accumulation of mutations hitchhiking with prominent resistance-associated mutations does not support the hypothesis of a stress induced mutational burst. However, the evolution of MTBC strains within the patient during drug treatment can be complex and mixtures of susceptible and resistant subpopulations as well as mixtures of subpopulations with different resistance conferring mutations can co-exist over long time periods. This diversity gives rise to MTBC strains which can adapt and evolve within the patient and therefore underlines the importance of a correct identification of mixed infections and treatment failures, arguing strongly for the application of genome based molecular epidemiology.

## Supporting Information

File S1 **Tables S1-S7.** The Tables S1-S7 include detailed results on genotypic and phenotypic drug resistance acquisition and applied regimens for Patient A, B and C. Individual tables of the filtered NGS data are given for each Patient as well as the combined table with all analyzed genome positions.
(XLSX)

Method S1 **A detailed description with examples for the estimation of proportions of clonal subpopualtions in single isolates utilizing the program VarDetect (http://www4a.biotec.or.th/GI/tools/vardetect).**
(DOCX)

## References

[1] WHO (2012) WHO | Tuberculosis Fact sheet. WHO. Available: http://www.who.int/mediacentre/factsheets/fs104/en/. Accessed 5 April 2012

[2] Cox HS, Sibilia C, Feuerriegel S, Kalon S, Polonsky J et al. (2008) Emergence of Extensive Drug Resistance during Treatment for Multidrug-Resistant. Tuberculosis - N Engl J Med 359: 2398–2400. doi:10.1056/NEJMc0805644. 19038891

[3] Ahuja SD, Ashkin D, Avendano M, Banerjee R, Bauer M et al. (2012) Multidrug resistant pulmonary tuberculosis treatment regimens and patient outcomes: an individual patient data meta-analysis of 9,153 patients. PLoS Med 9: e1001300. doi:10.1371/journal.pmed.1001300. PubMed: 22952439. 22952439 PMC3429397

[4] Borrell S, Gagneux S (2009) Infectiousness, reproductive fitness and evolution of drug-resistant Mycobacterium tuberculosis. Int J Tuberc Lung Dis 13: 1456–1466. PubMed: 19919762. 19919762

[5] De Steenwinkel JEM, ten Kate MT, de Knegt GJ, Kremer K, Aarnoutse RE et al. (2012) Drug susceptibility of Mycobacterium tuberculosis Beijing genotype and association with MDR TB. Emerg Infect Dis 18: 660–663. doi:10.3201/eid1804.110912. PubMed: 22469099. 22469099 PMC3309663

[6] Kubica T, Agzamova R, Wright A, Aziz MA, Rakishev G et al. (2005) The Beijing genotype is a major cause of drug-resistant tuberculosis in Kazakhstan. Int J Tuberc Lung Dis Off J Int Union Tuberc Lung Dis 9: 646–653. 15971392

[7] Cox HS, Kubica T, Doshetov D, Kebede Y, Rüsch-Gerdess S et al. (2005) The Beijing genotype and drug resistant tuberculosis in the Aral Sea region of Central Asia. Respir Res 6: 134. doi:10.1186/1465-9921-6-134. PubMed: 16277659. 16277659 PMC1299328

[8] Pardini M, Niemann S, Varaine F, Iona E, Meacci F et al. (2009) Characteristics of drug-resistant tuberculosis in Abkhazia (Georgia), a high-prevalence area in Eastern Europe. Tuberculosis (Edinb) 89: 317–324. doi:10.1016/j.tube.2009.04.002. PubMed: 19539531. 19539531

[9] Baranov AA, Mariandyshev AO, Mannsåker T, Dahle UR, Bjune GA (2009) Molecular epidemiology and drug resistance of widespread genotypes of Mycobacterium tuberculosis in northwestern Russia. Int J Tuberc Lung Dis 13: 1288–1293. PubMed: 19793435. 19793435

[10] Taddei F, Radman M, Maynard-Smith J, Toupance B, Gouyon PH et al. (1997) Role of mutator alleles in adaptive evolution. Nature 387: 700–702. doi:10.1038/42696. PubMed: 9192893. 9192893

[11] Sniegowski PD, Gerrish PJ, Lenski RE (1997) Evolution of high mutation rates in experimental populations of E. coli. Nature 387: 703–705. doi:10.1038/42701. PubMed: 9192894. 9192894

[12] Oliver A, Cantón R, Campo P, Baquero F, Blázquez J (2000) High frequency of hypermutable Pseudomonas aeruginosa in cystic fibrosis lung infection. Science 288: 1251–1254. doi:10.1126/science.288.5469.1251. PubMed: 10818002. 10818002

[13] Ford CB, Shah RR, Maeda MK, Gagneux S, Murray MB et al. (2013) Mycobacterium tuberculosis mutation rate estimates from different lineages predict substantial differences in the emergence of drug-resistant tuberculosis. Nat Genet 45: 784–790. doi:10.1038/ng.2656. PubMed: 23749189. 23749189 PMC3777616

[14] Werngren J, Hoffner SE (2003) Drug-susceptible Mycobacterium tuberculosis Beijing genotype does not develop mutation-conferred resistance to rifampin at an elevated rate. J Clin Microbiol 41: 1520–1524. doi:10.1128/JCM.41.4.1520-1524.2003. PubMed: 12682139. 12682139 PMC153924

[15] Roetzer A, Diel R, Kohl TA, Rückert C, Nübel U et al. (2013) Whole genome sequencing versus traditional genotyping for investigation of a Mycobacterium tuberculosis outbreak: a longitudinal molecular epidemiological study. PLoS Med 10: e1001387. doi:10.1371/journal.pmed.1001387. PubMed: 23424287. 23424287 PMC3570532

[16] Walker TM, Ip CLC, Harrell RH, Evans JT, Kapatai G et al. (2013) Whole-genome sequencing to delineate Mycobacterium tuberculosis outbreaks: a retrospective observational study. Lancet Infect Dis 13: 137–146. doi:10.1016/S1473-3099(12)70277-3. PubMed: 23158499. 23158499 PMC3556524

[17] Saunders NJ, Trivedi UH, Thomson ML, Doig C, Laurenson IF et al. (2011) Deep resequencing of serial sputum isolates of Mycobacterium tuberculosis during therapeutic failure due to poor compliance reveals stepwise mutation of key resistance genes on an otherwise stable genetic background. J Infect 62: 212–217. doi:10.1016/j.jinf.2011.01.003. PubMed: 21237201. 21237201

[18] Sun G, Luo T, Yang C, Dong X, Li J et al. (2012) Dynamic population changes in Mycobacterium tuberculosis during acquisition and fixation of drug resistance in patients. J Infect Dis, 206: 1724–33. doi:10.1093/infdis/jis601. PubMed: 22984115. 22984115 PMC3488197

[19] Niemann S, Richter E, Rüsch-Gerdes S, Schlaak M, Greinert U (2000) Double infection with a resistant and a multidrug-resistant strain of Mycobacterium tuberculosis. Emerg Infect Dis 6: 548–551. doi:10.3201/eid0605.000518. PubMed: 10998389. 10998389 PMC2627962

[20] Bonnet M, Pardini M, Meacci F, Orrù G, Yesilkaya H et al. (2011) Treatment of tuberculosis in a region with high drug resistance: outcomes, drug resistance amplification and re-infection. PLOS ONE 6: e23081. doi:10.1371/journal.pone.0023081. PubMed: 21886778. 21886778 PMC3160294

[21] Blom J, Jakobi T, Doppmeier D, Jaenicke S, Kalinowski J et al. (2011) Exact and complete short-read alignment to microbial genomes using Graphics Processing Unit programming. Bioinformatics 27: 1351–1358. doi:10.1093/bioinformatics/btr151. PubMed: 21450712. 21450712

[22] Ngamphiw C, Kulawonganunchai S, Assawamakin A, Jenwitheesuk E, Tongsima S (2008) VarDetect: a nucleotide sequence variation exploratory tool. BMC Bioinformatics 9 Suppl 12: S9. doi:10.1186/1471-2105-9-S12-S9. PubMed: 19091032. PMC2638149 19091032

[23] Ramaswamy SV, Reich R, Dou S-J, Jasperse L, Pan X et al. (2003) Single nucleotide polymorphisms in genes associated with isoniazid resistance in Mycobacterium tuberculosis. Antimicrob Agents Chemother 47: 1241–1250. doi:10.1128/AAC.47.4.1241-1250.2003. PubMed: 12654653. 12654653 PMC152487

[24] Da Silva PEA, Palomino JC (2011) Molecular basis and mechanisms of drug resistance in Mycobacterium tuberculosis: classical and new drugs. J Antimicrob Chemother 66: 1417–1430. doi:10.1093/jac/dkr173. PubMed: 21558086. 21558086

[25] Plinke C, Cox HS, Kalon S, Doshetov D, Rüsch-Gerdes S et al. (2009) Tuberculosis ethambutol resistance: concordance between phenotypic and genotypic test results. Tuberculosis (Edinb) 89: 448–452. doi:10.1016/j.tube.2009.09.001. PubMed: 19800845. 19800845

[26] Comas I, Borrell S, Roetzer A, Rose G, Malla B et al. (2012) Whole-genome sequencing of rifampicin-resistant Mycobacterium tuberculosis strains identifies compensatory mutations in RNA polymerase genes. Nat Genet 44: 106–110. doi:10.1038/ng.1038. PubMed: 22179134. PMC3246538 22179134

[27] Casali N, Nikolayevskyy V, Balabanova Y, Ignatyeva O, Kontsevaya I et al. (2012) Microevolution of extensively drug-resistant tuberculosis in Russia. Genome Res 22: 735–745. doi:10.1101/gr.128678.111. PubMed: 22294518. 22294518 PMC3317155

[28] DeBarber AE, Mdluli K, Bosman M, Bekker LG, Barry CE 3rd (2000) Ethionamide activation and sensitivity in multidrug-resistant Mycobacterium tuberculosis. Proc Natl Acad Sci U S A 97: 9677–9682. doi:10.1073/pnas.97.17.9677. PubMed: 10944230. 10944230 PMC16924

[29] Wang F, Langley R, Gulten G, Dover LG, Besra GS et al. (2007) Mechanism of thioamide drug action against tuberculosis and leprosy. J Exp Med 204: 73–78. doi:10.1084/jem.20062100. PubMed: 17227913. 17227913 PMC2118422

[30] Stoffels K, Mathys V, Fauville-Dufaux M, Wintjens R, Bifani P (2012) Systematic Analysis of Pyrazinamide-Resistant Spontaneous Mutants and Clinical Isolates of Mycobacterium tuberculosis. Antimicrob Agents Chemother. Available: http://aac.asm.org/content/early/2012/07/17/AAC.05385-11. Accessed 31 July 2012 10.1128/AAC.05385-11 PMC3457413 22825123

[31] Engström A, Morcillo N, Imperiale B, Hoffner SE, Juréen P (2012) Detection of First- and Second-Line Drug Resistance in Mycobacterium tuberculosis Clinical Isolates using Pyrosequencing. J Clin Microbiol. Available: http://www.ncbi.nlm.nih.gov/pubmed/22461677 Accessed 10 April 2012 10.1128/JCM.06664-11 PMC3372151 22461677

[32] Sun Z, Zhang J, Zhang X, Wang S, Zhang Y et al. (2008) Comparison of gyrA gene mutations between laboratory-selected ofloxacin-resistant Mycobacterium tuberculosis strains and clinical isolates. Int J Antimicrob Agents 31: 115–121. doi:10.1016/j.ijantimicag.2007.10.014. PubMed: 18164184. 18164184

[33] Lee H, Cho SN, Bang HE, Lee JH, Bai GH et al. (2000) Exclusive mutations related to isoniazid and ethionamide resistance among Mycobacterium tuberculosis isolates from Korea. Int J Tuberc Lung Dis 4: 441–447. PubMed: 10815738. 10815738

[34] Larsen MH, Vilchèze C, Kremer L, Besra GS, Parsons L et al. (2002) Overexpression of inhA, but not kasA, confers resistance to isoniazid and ethionamide in Mycobacterium smegmatis, M. bovis BCG and M. tuberculosis. Mol Microbiol 46: 453–466. doi:10.1046/j.1365-2958.2002.03162.x. PubMed: 12406221. 12406221

[35] Cavusoglu C, Karaca-Derici Y, Bilgic A (2004) In-vitro activity of rifabutin against rifampicin-resistant Mycobacterium tuberculosis isolates with known rpoB mutations. Clin Microbiol Infect 10: 662–665. doi:10.1111/j.1469-0691.2004.00917.x. PubMed: 15214882. 15214882

[36] Ramaswamy SV, Amin AG, Göksel S, Stager CE, Dou SJ et al. (2000) Molecular genetic analysis of nucleotide polymorphisms associated with ethambutol resistance in human isolates of Mycobacterium tuberculosis. Antimicrob Agents Chemother 44: 326–336. doi:10.1128/AAC.44.2.326-336.2000. PubMed: 10639358. 10639358 PMC89679

[37] Mathys V, Wintjens R, Lefevre P, Bertout J, Singhal A et al. (2009) Molecular genetics of para-aminosalicylic acid resistance in clinical isolates and spontaneous mutants of Mycobacterium tuberculosis. Antimicrob Agents Chemother 53: 2100–2109. doi:10.1128/AAC.01197-08. PubMed: 19237648. 19237648 PMC2681553

[38] Schürch AC, Kremer K, Kiers A, Daviena O, Boeree MJ et al. (2010) The tempo and mode of molecular evolution of Mycobacterium tuberculosis at patient-to-patient scale. Infect Genet Evol 10: 108–114. doi:10.1016/j.meegid.2009.10.002. PubMed: 19835997. 19835997

[39] Post FA, Willcox PA, Mathema B, Steyn LM, Shean K et al. (2004) Genetic polymorphism in Mycobacterium tuberculosis isolates from patients with chronic multidrug-resistant tuberculosis. J Infect Dis 190: 99–106. doi:10.1086/421501. PubMed: 15195248. 15195248

[40] Ford CB, Lin PL, Chase MR, Shah RR, Iartchouk O et al. (2011) Use of whole genome sequencing to estimate the mutation rate of Mycobacterium tuberculosis during latent infection. Nat Genet 43: 482–486. doi:10.1038/ng.811. PubMed: 21516081. 21516081 PMC3101871

[41] Heep M, Brandstätter B, Rieger U, Lehn N, Richter E et al. (2001) Frequency of rpoB Mutations Inside and Outside the Cluster I Region in Rifampin-Resistant Clinical Mycobacterium tuberculosis Isolates. J Clin Microbiol 39: 107–110. doi:10.1128/JCM.39.1.107-110.2001. PubMed: 11136757. 11136757 PMC87688

[42] Streicher EM, Bergval I, Dheda K, Böttger EC, Gey van Pittius NC et al. (2012) Mycobacterium tuberculosis population structure determines the outcome of genetics-based second-line drug resistance testing. Antimicrob Agents Chemother 56: 2420–2427. doi:10.1128/AAC.05905-11. PubMed: 22330913. 22330913 PMC3346650

[43] Zhang Y, Yew WW (2009) Mechanisms of drug resistance in Mycobacterium tuberculosis. Int J Tuberc Lung Dis Off J Int Union Tuberc Lung Dis 13: 1320–1330. 19861002

[44] Fivian-Hughes AS, Houghton J, Davis EO (2012) Mycobacterium tuberculosis thymidylate synthase gene thyX is essential and potentially bifunctional, while thyA deletion confers resistance to p-aminosalicylic acid. Microbiology 158: 308–318. doi:10.1099/mic.0.053983-0. PubMed: 22034487. 22034487 PMC3352284

[45] Zhang Y (2005) The magic bullets and tuberculosis drug targets. Annu Rev Pharmacol Toxicol 45: 529–564. doi:10.1146/annurev.pharmtox.45.120403.100120. PubMed: 15822188. 15822188

[46] Cáceres NE, Harris NB, Wellehan JF, Feng Z, Kapur V et al. (1997) Overexpression of the D-alanine racemase gene confers resistance to D-cycloserine in Mycobacterium smegmatis. J Bacteriol 179: 5046–5055. PubMed: 9260945. 9260945 10.1128/jb.179.16.5046-5055.1997 PMC179361

[47] Niemann S, Köser CU, Gagneux S, Plinke C, Homolka S et al. (2009) Genomic diversity among drug sensitive and multidrug resistant isolates of Mycobacterium tuberculosis with identical DNA fingerprints. PLOS ONE 4: e7407. doi:10.1371/journal.pone.0007407. PubMed: 19823582. 19823582 PMC2756628

