# Supplementary material for: Whole Genome Sequencing Reveals Complex Evolution Patterns of Multidrug-Resistant Mycobacterium tuberculosis Beijing Strains in Patients
Source: PLoS One. 2013 Dec 6;8(12):e82551. doi: 10.1371/journal.pone.0082551 (PMC3855793; doi:10.1371/journal.pone.0082551)
Supplement: Method S1 — A detailed description with examples for the estimation of proportions of clonal subpopualtions in single isolates utilizing the program VarDetect (http://www4a.biotec.or.th/GI/tools/vardetect). (DOCX) [file pone.0082551.s002.docx]

**Supplementary Method S1**

**Estimating the proportion of different clonal subpopulation in a single isolate**

Based on the targets identified with the WGS approach and screening of drug resistance mediating genes of multiple (up to 15) serial isolates we identified characteristic SNPs for subpopulations dominating the intra patient bacterial population during the treatment period. Alternating patterns of polymorphisms (see Supplementary table S2 and S4 for Patient A and B, respectively) already indicate the presence, absence or mixture of different clones in single isolates. To quantify this we applied VarDetect (vers. 200601251500) [1] to analyze the peak height ratios for heterogeneous variant calls. If more than two variants were present in a single isolate we logically inferred the proportions as in the following examples:

**Patient A on month 15 (see Figure 1A below):**

1. The variation in Rv0565c is linked to the deletion of *pncA* in A3 (table S2 and comparing chromatograms and PCR results, as well as drop in coverage in the *pncA* region in the NGS data from the isolate on month 26 coinciding with the high frequency SNP in *Rv0565c* R59H, Pos# 657.295 in table S1).
2. On month 15 the SNP leading to *Rv0565c* R59H had a proportion of 50% at the given position in the chromatogram. Knowing that the SNPs in *pncA* cannot be attributed to the *pncA* deleted clone A3, the other half must reflect different variants.
3. The SNP *pncA* L159V (clone A1) was only detected on month 15, whereas the SNP *pncA* T100P (clone A2) was present till month 36. Thus we conclude that these are two different clones and both variant calls confirm a ratio of 70:30 (A1:A2), that is in total 35% A1, 15% A2 and 50% A3.


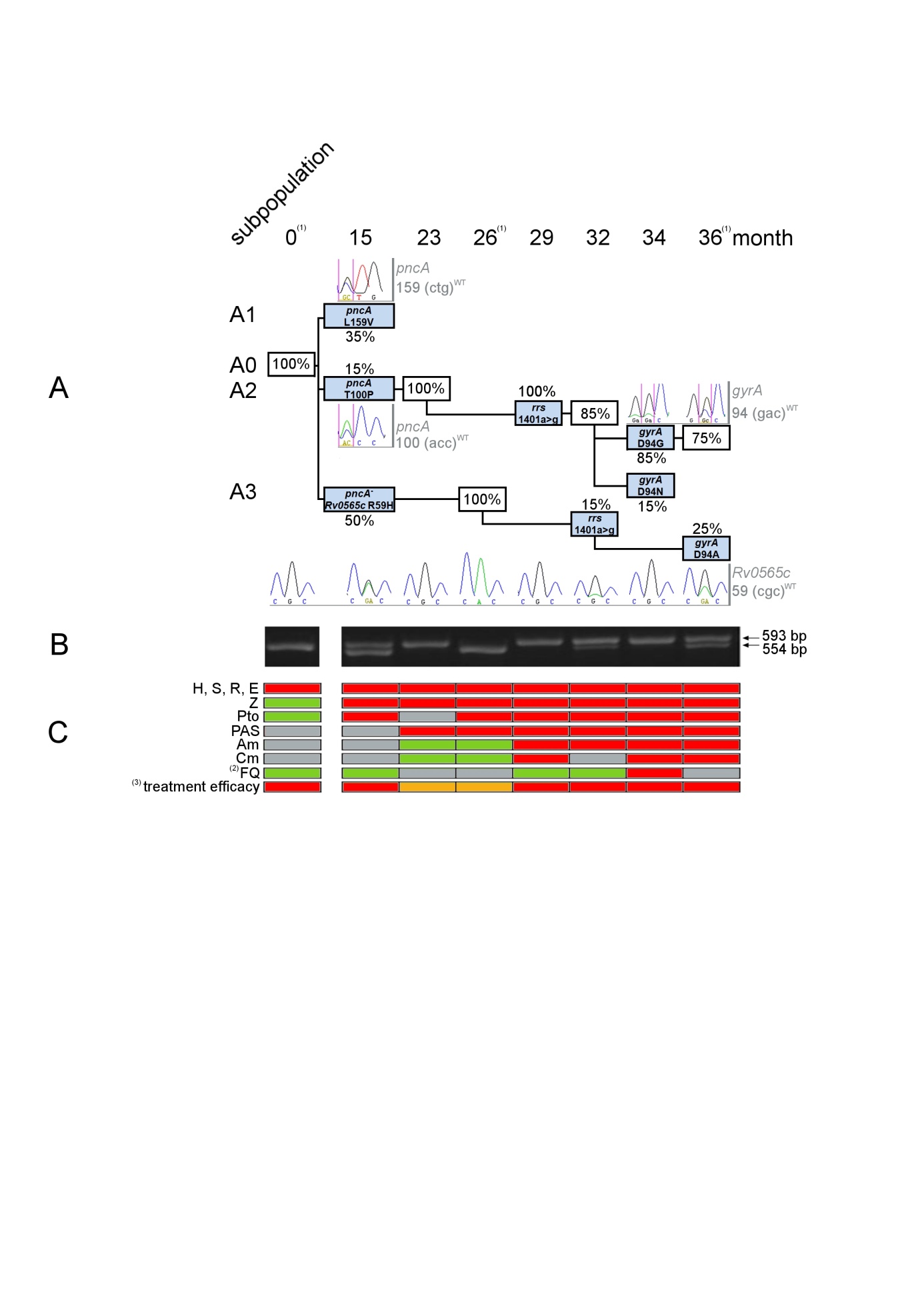


**Patient B on month 8 (see Figure 2A below)**

1. The alternating pattern in *rpoB* mutations A435G and A584G (table S4) indicates the presence of at least two different clones.
2. On month 8 we could in addition detect multiple variants for *gyrA* in codon 94 (KRC) that can be translated to GAC (wt), GGC, TAC, TGC and *rpoB* wt and A584G, respectively.
3. The *gyrA* mutations were linked to *rpoB* A584G taken the follow up isolates into account, only showing this combination together untill month 21.
4. The ratio of *rpoB* WT to *rpoB* A584G was 20:80, and consequently the wt *gyrA* GAC then also should have 20%. TAC is definitely an existing codon that could be detected on month 12 uniquely. GGC and TGC can be possible other variants on month 8.
5. The proportion of the Guanin in the first codon position to Thymin is 80:20, whereas 20% is attributed to the wt codon GAC, thus 60% must be attributed to the codon GGC. The remaining 20% is a signal from the clone carrying TAC (TGC is most likely not in the mixture, because the ratio of Guanin to Adenin at the second codon position is not exceeding 50:50)


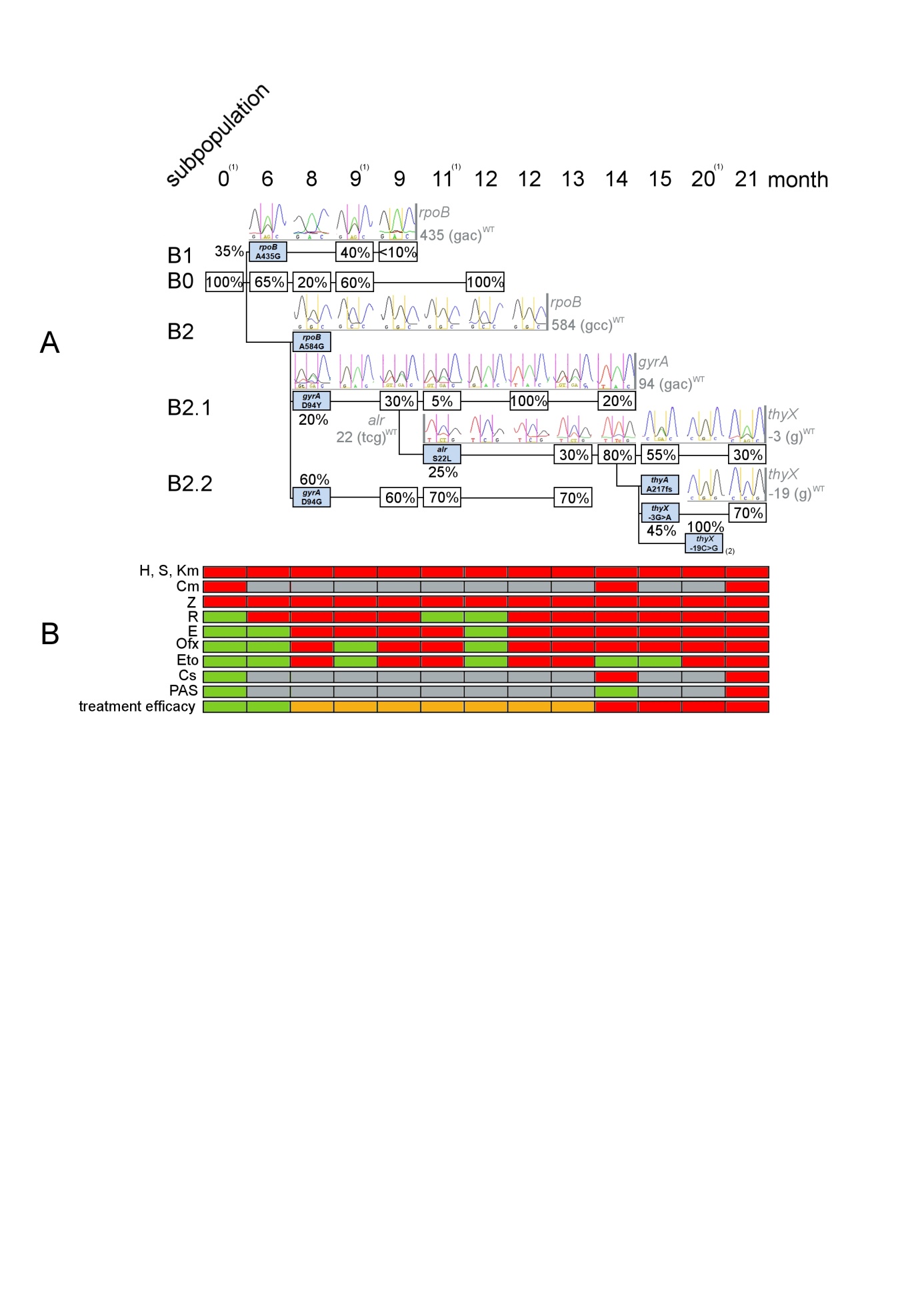


1. Ngamphiw C, Kulawonganunchai S, Assawamakin A, Jenwitheesuk E, Tongsima S (2008) VarDetect: a nucleotide sequence variation exploratory tool. BMC Bioinformatics 9 Suppl 12: S9. doi:10.1186/1471-2105-9-S12-S9.
